# Structure-based comparison and functional verification reveal LEF-3 as a novel core gene of baculoviruses

**DOI:** 10.1128/jvi.00230-26

**Published:** 2026-09-29

**Authors:** Hengrui Hu, Xinyu Zhang, Xinyu Tian, Jiang Li, Zhihong Hu, Manli Wang

**Affiliations:** 1 State Key Laboratory of Virology and Biosafety, Wuhan Institute of Virology, Chinese Academy of Sciences 74614, Wuhan, Hubei, China; Wageningen University & Research, Wageningen, Netherlands

**Keywords:** baculovirus, core gene, LEF-3, structure prediction, homology search

## LETTER

Baculoviruses comprise a diverse family of large DNA viruses categorized into four genera: *Alphabaculovirus*, *Betabaculovirus*, *Gammabaculovirus*, and *Deltabaculovirus* (1). While comparative genomics has previously identified 38 conserved core genes across all baculoviruses, the extreme sequence divergence of *Gammabaculovirus* and *Deltabaculovirus* lineages relative to the lepidopteran-infecting baculoviruses has hindered the identification of additional conserved components using traditional sequence alignment methods (2–4). Late expression factor 3 (LEF-3) is a multifaceted single-stranded DNA-binding protein (SSB) that not only stabilizes replication intermediates but also acts as a molecular chaperone for the viral helicase, P143, facilitating its essential nuclear localization (5–8). Despite its indispensable role in the baculovirus life cycle, LEF-3 was previously believed to be conserved only within the *Alphabaculovirus* and *Betabaculovirus* genera (3, 4).

**Fig 1:**
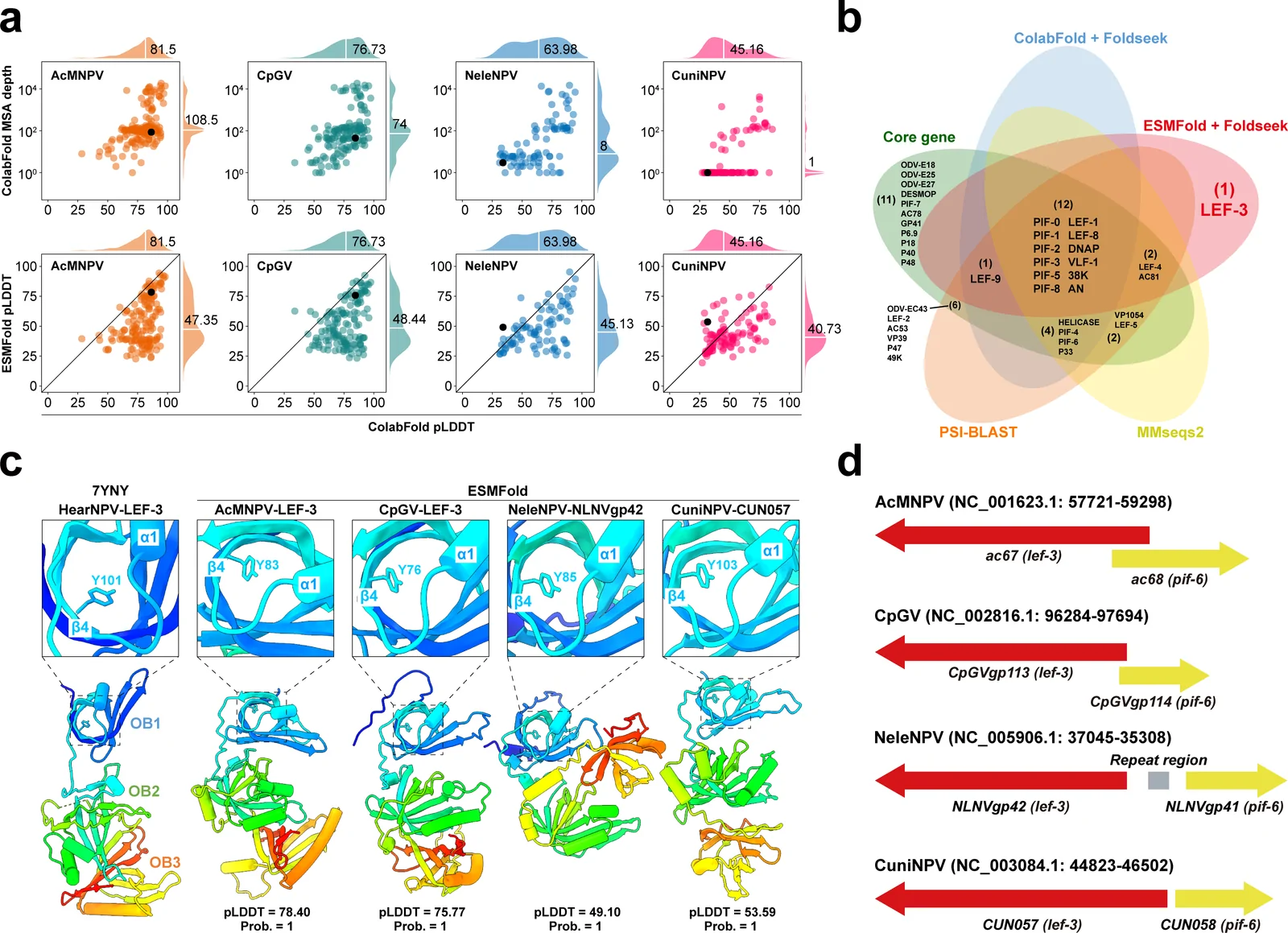
Structural prediction and alignment reveal LEF-3 conservation across all baculovirus genera. (**a**) Performance comparison of structure prediction methods in four representative baculoviruses. Upper panel: correlation between pLDDT scores and MSA depth for ColabFold predictions. Lower panel: pLDDT confidence scores for ColabFold vs ESMFold. Each point represents a single protein; the ridge plot shows the distribution density, with the median marked by a white line and the number. Homologs of LEF-3 are marked in black. (**b**) Venn diagram comparing baculovirus core genes with homologs identified by sequence- and structure-based methods. The gene intersections were labeled. Previously hidden conservation homologs of LEF-3 are identified by Foldseek based on ESMFold predicted structures. (**c**) Structural alignment of LEF-3 homologs. HearNPV LEF-3 structure is from PDB 7YNY; others are ESMFold-predicted. Insets highlight the conserved Y101 residue on the fourth β-strand of the OB1 domain. Colors transition from N-terminus (blue) to C-terminus (red). (**d**) Genomic maps illustrating the conserved synteny of *lef-3* and *pif-6* across genera.

In this study, we employed ColabFold and ESMFold to predict the protein structures of four representative baculoviruses—Autographa californica multiple nucleopolyhedrovirus (AcMNPV, *Alphabaculovirus*), Cydia pomonella granulovirus (CpGV, *Betabaculovirus*), Neodiprion lecontei nucleopolyhedrovirus (NeleNPV, *Gammabaculovirus*), and Culex nigripalpus nucleopolyhedrovirus (CuniNPV, *Deltabaculovirus*) (9, 10). While ColabFold yielded higher predicted local distance difference test (pLDDT) scores for viruses with rich multiple sequence alignments (MSAs), its performance significantly decreased for CuniNPV due to the scarcity of homologous sequences available for this sole *Deltabaculovirus* member (Fig. 1a). Conversely, the MSA-independent ESMFold provided superior structural predictions for multiple CuniNPV proteins, highlighting its value for modeling highly divergent or “orphan” viral lineages.

By leveraging the sequences and structures of these four representative viruses, structural clustering of ESMFold-predicted models via Foldseek unmasked previously hidden conservation. This approach successfully identified LEF-3 homologs in both NeleNPV (protein NLNVgp42) and CuniNPV (protein CUN057), both of which had eluded detection by PSI-BLAST, MMseqs, and previously exhaustive sequence-based search strategies (3) (Fig. 1b). Recently, the crystal structure of LEF-3 of Helicoverpa armigera nucleopolyhedrovirus (HearNPV) was resolved and it exhibits a characteristic architecture of three oligonucleotide-binding fold domains (OB1–OB3) (5). Our predicted structures for NLNVgp42 and CUN057 aligned closely with HearNPV LEF-3 (HaLEF-3), preserving the distinct OB1–OB3 structure with a Foldseek homology probability of 1. Furthermore, structure-guided sequence alignment identified strictly conserved residues, most notably a tyrosine (Y101 in HaLEF-3) within the OB1 domain (Fig. 1c; Fig. S1). Critically, genomic synteny analysis showed that *lef-3*, *NLNVgp42*, and *CUN057* all reside upstream of *pif-6*, another core gene, across all four genera. This combined evidence of structural homology and conserved genomic context strongly supports the designation of NLNVgp42 and CUN057 as bona fide LEF-3 orthologs (Fig. 1d).

**Fig 2:**
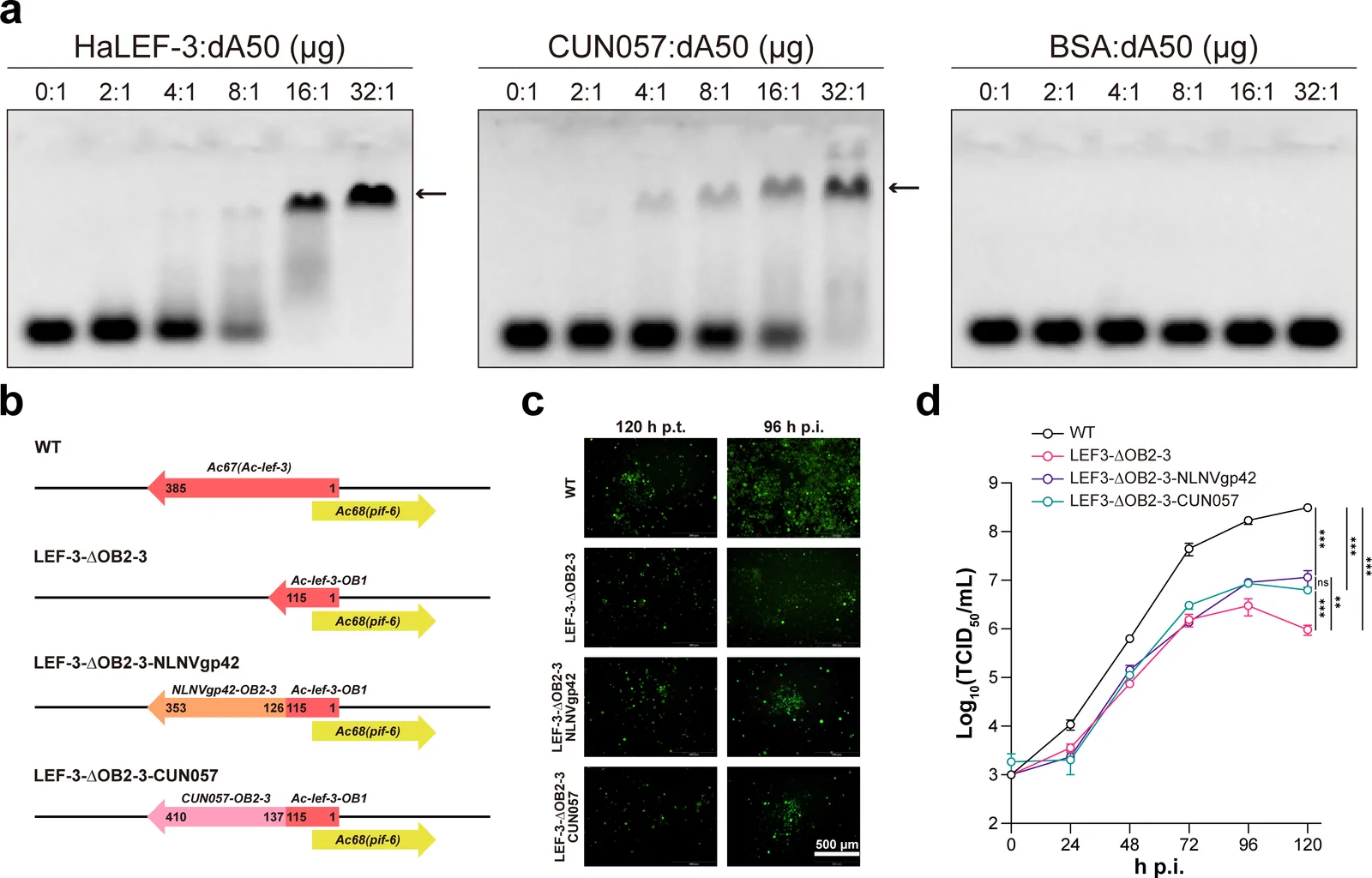
Functional characterization of HaLEF-3, NLNVgp42, and CUN057. (**a**) EMSA showing binding of HaLEF-3, CUN057, and BSA control to a 50 bp poly(A) ssDNA substrate at different ratios. The arrows indicate the shifted bands. (**b**) Schematic of LEF-3 truncation and domain-substitution constructs. (**c**) Fluorescence microscopy of the corresponding virus in panel b. Cells were imaged at 120 h post-transfection (h p.t.) and 96 h post-infection (h p.i.). (**d**) Growth curves of different recombinant viruses in Sf9 cells (MOI = 0.5). Data were obtained from three biological replicates and are represented as mean ± SD (*n* = 3); Tukey’s multiple comparison test was used to determine the statistical differences. **P < 0.01 and ***P < 0.001. ns, not significant.

To validate functional conservation, we expressed and purified CUN057 and the canonical HaLEF-3 using the baculovirus and prokaryotic expression systems, respectively (Fig. S2). Electrophoretic mobility shift assays (EMSAs) demonstrated that both HaLEF-3 and CUN057 bind ssDNA in a dose-dependent manner (Fig. 2a). Functional studies of AcMNPV LEF-3 indicate that the N-terminal OB1 domain mediates nuclear localization and helicase interaction, which is indispensable for viral replication, while the C-terminal OB2 and OB3 domains are involved in DNA binding, and their deletion leads to a significant reduction in viral titers (5–7, 11, 12). Using an AcMNPV synthetic platform (13, 14), we constructed a mutant lacking the OB2-3 domains and replaced this region with the corresponding segments from either NLNVgp42 or CUN057 (Fig. 2b). Fluorescence imaging confirmed that although the deletion mutant exhibited severely impaired replication, the domain-swapped recombinants remained capable of producing viable progeny (Fig. 2c). Growth curve analysis further demonstrated that these chimeric viruses reached significantly higher titers than the deletion mutant (Fig. 2d). These results indicate that the OB-fold domains from NeleNPV and CuniNPV LEF-3 homologs retain functional compatibility with that of AcMNPV during virus infection.

In summary, by integrating AI-driven structural prediction with biochemical and virological validation, we establish LEF-3 as a novel core gene of the *Baculoviridae*. Despite their low sequence identity, which eluded detection by BLAST and MMseqs, CUN057 and NLNVgp42 exhibited clear structural similarity to LEF-3. This study highlights the limitations of sequence-centric genomics in studying highly divergent viruses. Our findings suggest that the need for an OB-fold-based SSB to stabilize the viral genome is a conserved requirement. Interestingly, large-scale viral protein structural alignment revealed the widespread presence of similar OB-fold proteins in poxviruses and bacteriophages (15), indicating that structural comparison is a useful tool for revealing the “dark matter” of large DNA virus families.

## SUPPLEMENTAL MATERIAL

Supplemental material jvi.00230-26-s0001.pdf Fig. S1 and S2, Table S1, and supplemental methods.

ASM does not own the copyrights to Supplemental Material that may be linked to, or accessed through, an article. The authors have granted ASM a non-exclusive, world-wide license to publish the Supplemental Material files. Please contact the corresponding author directly for reuse.
