# Supplementary material for: Structure-based comparison and functional verification reveal LEF-3 as a novel core gene of baculoviruses
Source: J Virol. 2026 Sep 29;100(10):e00230-26. doi: 10.1128/jvi.00230-26 (PMC13712923; doi:10.1128/jvi.00230-26)
Supplement: Supplementary file 1 [file jvi.00230-26-s0001.pdf]

Supplemental Material

Structure-based comparison and functional verification reveal LEF-3 as a novel core gene of baculoviruses

Hengrui Hu[1], Xinyu Zhang[1], Xinyu Tian[1], Jiang Li[1], Zhihong Hu[1*], Manli Wang[1*]

1. State Key Laboratory of Virology and Biosafety, Wuhan Institute of Virology, Chinese Academy of Sciences, Wuhan, Hubei, China

* Co-corresponding authors:

Manli Wang, Dr. Prof. Mailing address: Wuhan Institute of Virology, Chinese Academy of Sciences, Wuhan 430071, P.R. China.

Email: wangml@wh.iov.cn

Zhihong Hu, Dr. Prof. Mailing address: Wuhan Institute of Virology, Chinese Academy of Sciences, Wuhan 430071, P.R. China.

Email: huzh@wh.iov.cn

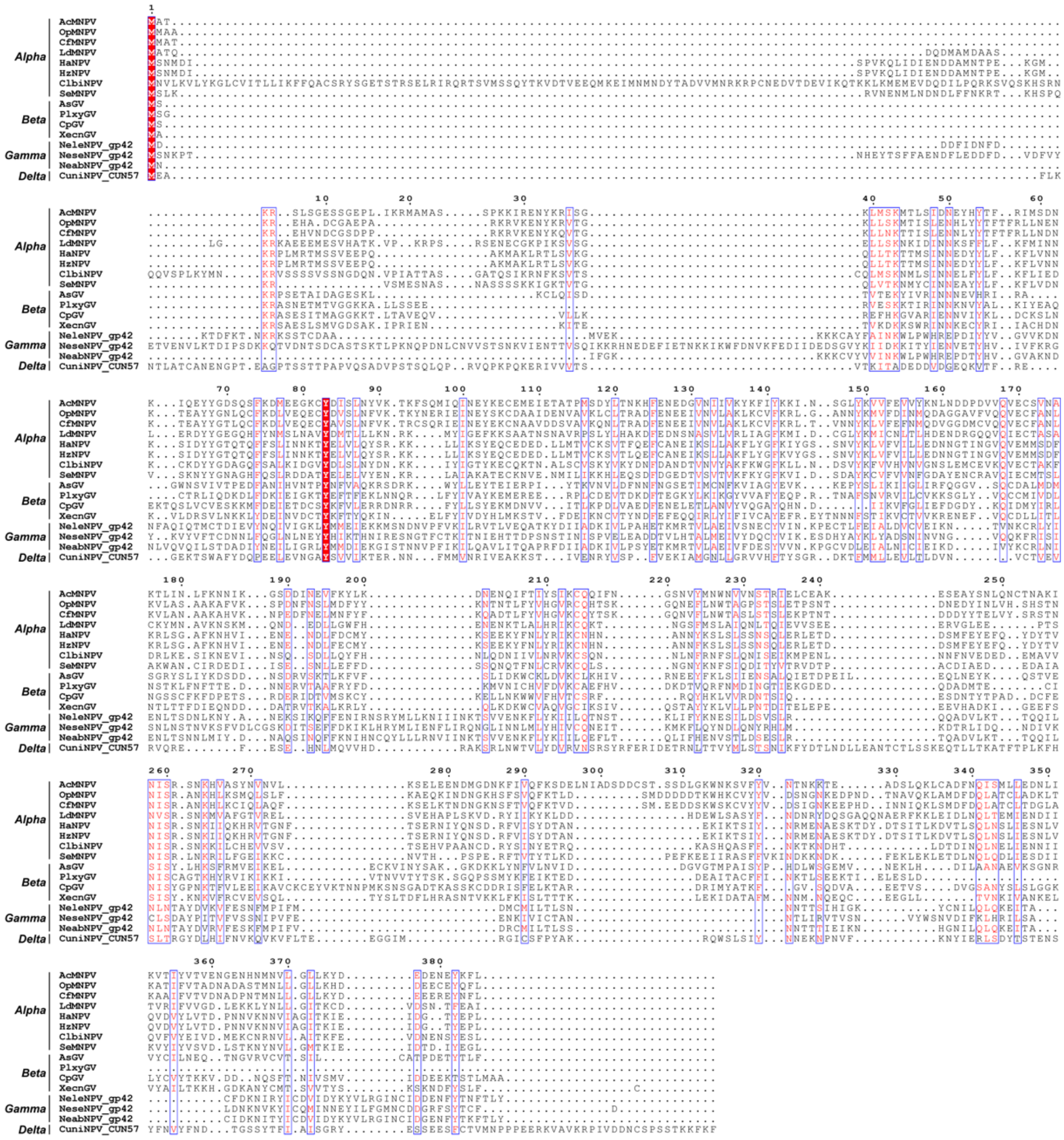

Figure S1. Sequence alignment of LEF-3 homologs across four baculovirus genera.

Homologous LEF-3 sequences were aligned using MAFFT (https://toolkit.tuebingen.mpg.de/tools/mafft) and visualized with ESPript 3.0 (https://espript.ibcp.fr/ESPript/ESPript/index.php). Conserved residues are highlighted, with the strictly conserved Y101 residue noted in the main text.

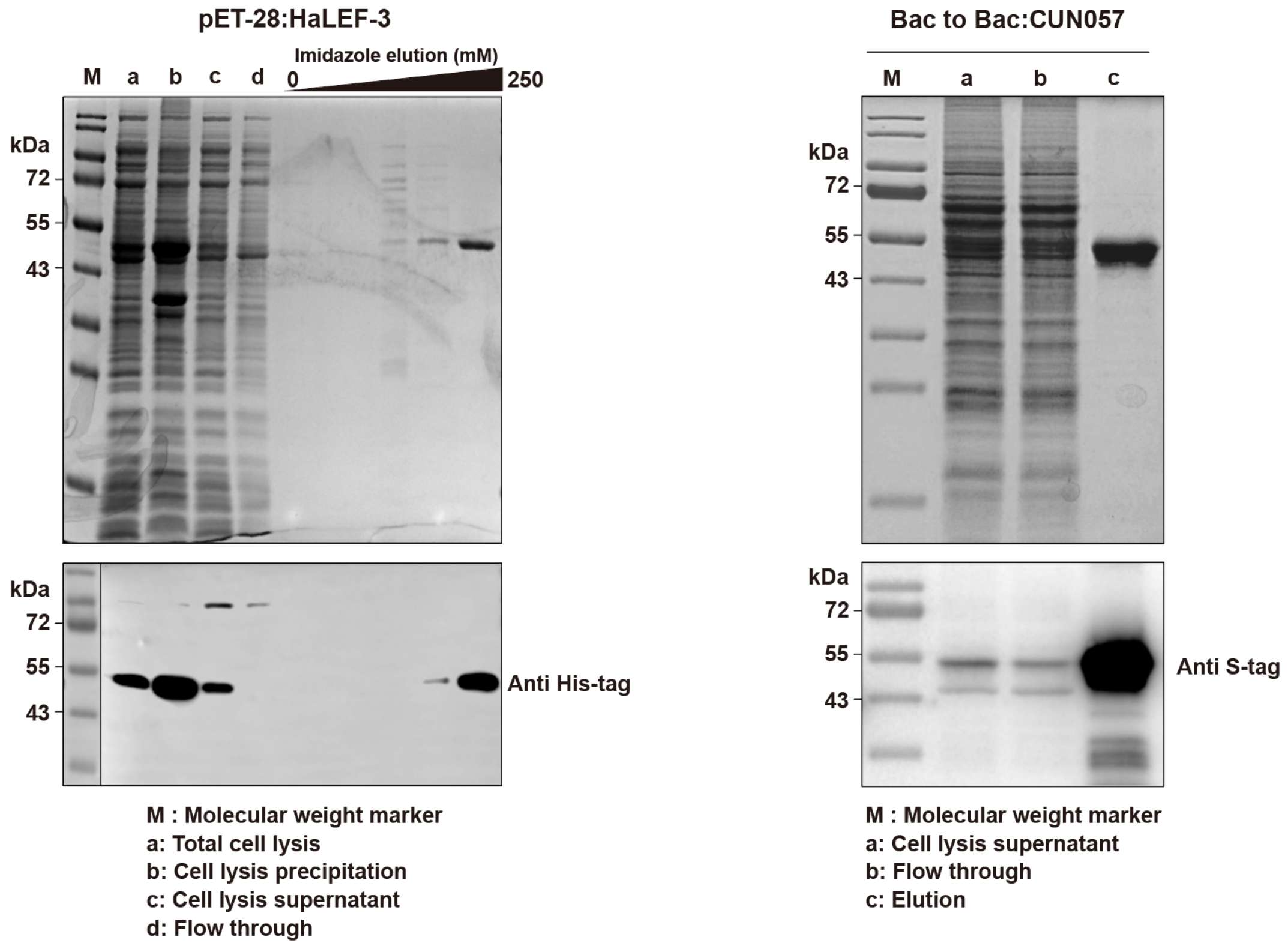

Figure S2. SDS-PAGE and western blot analysis of purified HaLEF-3 and CUN057

HaLEF-3 was cloned into pET-28a vector and purified using Ni-NTA agarose column. CUN057 were cloned into pFBD vector and then recombinant into baculovirus; the proteins were purified using S-protein resin. In SDS-PAGE analysis, the proteins were resolved on a 12% SDS-PAGE gel and stained with Coomassie Brilliant Blue to assess purity and molecular weight. In Western blot analysis, antibodies against His-tag and S-tag were used to recognize the target proteins. Components in different lanes are labeled below the figure.

Table S1. Primers used in this study

Primer	Sequence
LEF3-OB1-R	gggggttgcagtttcaatttccattt
pALEF3-OB1-F	tgtgaaatggaaattgaaactgcaacccccctaattctattcgacgtttggttgaacgct
gp42-OB1-F	aaagaatgtgaaatggaaattgaaactgcaaccccccgcgcacgaaacaaaaatgcgtac
gp42-pA-R	aaccaaacgtcgaatagaattaatacaacgtaaaatttgtataaaagttttcatcgtcg
pALEF3-F	taattctattcgacgtttggttgaacgct
CUN057-OB1-F	aaagaatgtgaaatggaaattgaaactgcaacccccctttgtagagaaaattgcgatggg
CUN057-pA-R	gccagcgttcaaccaaacgtcgaatagaattaaaatttaaactttttggtggagctggg

Materials and methods

Cells and Plasmids

Sf9 insect cells were cultured at 27 °C in Grace's medium (Invitrogen, USA) supplemented with 10% fetal bovine serum (FBS) (Gibco, USA). The Saccharomyces cerevisiae strain VL6−48 and the TAR cloning vector pGF were kindly provided by Prof. Gengfu Xiao (Wuhan Institute of Virology, CAS). The pET-28a–HaLEF-3 expression plasmid was provided by Prof. Sheng Cao (Wuhan Institute of Virology, CAS). Plasmids containing partitioned AcMNPV genome fragments for virus rescue were constructed and maintained in our laboratory as previously described.

Protein Structure Prediction and Homology Search

Coding sequences for AcMNPV (NC_001623.1), CpGV (NC_002816.1), NeleNPV (NC_005906.1), and CuniNPV (NC_003084.1) were retrieved from the NCBI database. Protein structures were predicted using LocalColabFold (1) and ESMFold (2) with default settings. Multiple sequence alignments (MSAs) for LocalColabFold are generated by searching the UniRef30, ColabFold_envdb, and pdb100 databases (https://opendata.mmseqs.org/colabfold) locally using MMseqs2 (5). The top-ranked model for each protein was selected for downstream analysis. An "all-against-all" structural search was performed using Foldseek (3). Structural homologs were identified using a probability threshold (prob) > 0.9. For sequence-based searches, PSI-BLAST (4) and MMseqs2 were employed with an E-value cutoff of 0.001.

Protein Expression and Purification

HaLEF-3 was expressed in *E. coli* BL21(DE3) cells as previously described (6). Briefly, cultures were grown at 37 °C to an OD_{600} of 0.6–0.8, induced with 0.5 mM IPTG, and incubated at 16 °C for 18 h. Cells were harvested, lysed via sonication in lysis buffer (50 mM NaCl, 20 mM imidazole, 50 mM Tris–HCl, pH 7.5), and the supernatant was purified using a Ni-NTA agarose column (TransGen, China). The protein was eluted with 250 mM imidazole and further concentrated using 30 kDa MWCO centrifugal filters (Millipore).

CUN057 were expressed using the Bac-to-Bac baculovirus expression system. Coding sequences were N-terminally tagged with an S-Tag, synthesized, and cloned into the pFBD vector (Tsingke, China). Recombinant baculoviruses were amplified in Sf9 cells at a low multiplicity of infection (MOI = 0.02). For protein production, Sf9 cells were infected at an MOI of 5, harvested 96 h post-infection, and lysed. Target proteins were purified using S-protein resin (Millipore, Germany) and eluted by 3 M $MgCl_2$ solution according to the manufacturer's protocol. The elution was concentrated and replaced in lysis buffer using 30 kDa MWCO centrifugal filters (Millipore). Protein concentrations were determined by the Bradford assay (Beyotime, China).

Electrophoretic Mobility Shift Assay (EMSA)

Synthetic oligo-deoxyadenosine (dA_{50}) substrates with 6-carboxy-fluorescein labeled at both the 5′ and 3′ ends were purchased from Sangon Biotech. In 10 µL reactions, varying amounts of protein (HaLEF-3, CUN057, or BSA) were mixed with ssDNA at

protein-to-DNA mass ratios of 0:1, 1:1, 2:1, 4:1, 8:1, 16:1, and 32:1. Mixtures were incubated at room temperature for 60 min. Bound and unbound DNA were separated on a 1% agarose gel in 1× TAE buffer. Fluorescein-labeled ssDNA and ssDNA–SSB complexes were visualized via 488 nm excitation light (Azure 280, USA).

Construction and Rescue of Chimeric Viruses

Recombinant AcMNPV carrying LEF-3 functional complementation constructs were generated using a synthetic genomics platform (7, 8). The AcMNPV genome was partitioned into B-level fragments (B1–B9) and assembled into C-level fragments (C1–C3); *lef-3* is located within fragment B4. Three modified B4 constructs were generated via PCR (primers are listed in Table S1) and *in vitro* recombination (ClonExpress Ultra, Vazyme): (i) an OB2–OB3 deletion mutant (pGF-B4-ΔOB2-3), (ii) a complementation construct with the NLNVgp42 OB2–3 domain, and (iii) a complementation construct with the CUN057 OB2–3 domain.

For virus rescue, Sf9 cells were co-transfected with modified B4 fragments and the necessary supporting fragments (C1, B5, B6, and C3-*egfp*) using Cellfectin (Gibco). At 120 h post-transfection, supernatants were passaged once in Sf9 cells to amplify the virus. Supernatants were harvested 96 h post-passage, clarified by centrifugation, and titers were determined via endpoint dilution assay (EPDA).

Growth Curve Analysis

Sf9 cells (1×10^6 cells/well) were infected with recombinant viruses at an MOI of 0.1.

Supernatants were collected at 0, 24, 48, 72, 96, and 120 h post-infection (p.i.). Viral titers were determined by EPDA in triplicate, and data are presented as mean $TCID_{50}$/mL ± standard deviation (SD).

Reference

1. Mirdita M, Schutze K, Moriwaki Y, Heo L, Ovchinnikov S, Steinegger M. 2022. ColabFold: making protein folding accessible to all. Nat Methods 19:679–682.
2. Lin Z, Akin H, Rao R, Hie B, Zhu Z, Lu W, Smetanin N, Verkuil R, Kabeli O, Shmueli Y, Dos Santos Costa A, Fazel-Zarandi M, Sercu T, Candido S, Rives A. 2023. Evolutionary-scale prediction of atomic-level protein structure with a language model. Science 379:1123–1130.
3. van Kempen M, Kim SS, Tumescheit C, Mirdita M, Lee J, Gilchrist CLM, Soding J, Steinegger M. 2024. Fast and accurate protein structure search with Foldseek. Nat Biotechnol 42:243–246.
4. Altschul SF, Madden TL, Schaffer AA, Zhang J, Zhang Z, Miller W, Lipman DJ. 1997. Gapped BLAST and PSI-BLAST: a new generation of protein database search programs. Nucleic Acids Res 25:3389–402.
5. Steinegger M, Soding J. 2017. MMseqs2 enables sensitive protein sequence searching for the analysis of massive data sets. Nat Biotechnol 35:1026–1028.
6. Yin J, Fu Y, Rao G, Li Z, Tian K, Chong T, Kuang K, Wang M, Hu Z, Cao S. 2022. Structural transitions during the cooperative assembly of baculovirus single-stranded DNA-binding protein on ssDNA. Nucleic Acids Res 50:13100–13113.
7. Guo Y, Hu H, Xiao H, Wang X, Ke X, Li J, Wang M, Hu Z. 2025. Genome-Wide Simplification of the AcMNPV Genome Using Synthetic Biology. ACS Synth Biol 14:3414–3422.
8. Shang Y, Wang M, Xiao G, Wang X, Hou D, Pan K, Liu S, Li J, Wang J, Arif BM, Vlak JM, Chen X, Wang H, Deng F, Hu Z. 2017. Construction and Rescue of a Functional Synthetic Baculovirus. ACS Synth Biol 6:1393–1402.
